# Changes in residents’ hygiene awareness and behaviors in public toilets before and during the COVID-19 pandemic in Hangzhou, China: a two-round cross-sectional study

**DOI:** 10.1186/s12889-022-14114-8

**Published:** 2022-09-06

**Authors:** Jiayao Xu, Xin Xu, Kai Sing Sun, Dan Wu, Tai Pong Lam, Xudong Zhou

**Affiliations:** 1 Institute of Social Medicine, School of Medicine, Zhejiang University, 866 Yuhangtang Road, Hangzhou, China; 2 JC School of Public Health and Primary Care, The Chinese University of Hong Kong, Hong Kong, China; 3 Department of Clinical Research, London School of Hygiene & Tropical Medicine, London, UK; 4 Department of Family Medicine and Primary Care, The University of Hong Kong, Pokfulam, Hong Kong China; 5 The Second Affiliated Hospital, Zhejiang University School of Medicine, Hangzhou, China

**Keywords:** Hygiene behaviors, COVID-19, Hand hygiene, Public toilets, Hygiene amenities

## Abstract

**Background:**

Hygiene behaviors in public toilets are important to prevent the transmission of infectious diseases, especially during the pandemic. All through the novel coronavirus (COVID-19) pandemic, governments in many countries published guidance on personal hygiene for the general population to prevent disease transmission. This study aimed to investigate improvements in residents’ hygiene awareness and behaviors in public toilets before and during the pandemic.

**Methods:**

We recruited 316 residents between November and December 2018 before the pandemic, and 314 residents between December 2020 and January 2021 during the pandemic in the same study sites in Hangzhou, a well-developed city in China. Residents’ hygiene behaviors in public toilets, hygiene awareness, risk perception, and sociodemographic factors were collected. Bivariate analysis and multivariable logistic regressions were used to test the differences between the two rounds. We conducted an observational study to record the provision of hygiene amenities at toilets during the pandemic.

**Results:**

After controlling for sociodemographic factors (gender, marital status, age, education level, and monthly household income), compared with respondents recruited before the pandemic, respondents recruited during the pandemic were more likely to perceive the risks of infection when using public toilets (aOR = 1.77, 95%CI [1.20, 2.60]), and were more likely to be aware of the risks of touching contaminated toilet facilities (aOR = 1.72, 95%CI [1.17, 2.54]) and the risks of not using soap to wash one’s hands after using the toilet (aOR = 1.93, 95%CI [1.38, 2.72]). They were more likely to always clean their toilet seat with alcohol (aOR = 1.88, 95%CI [1.01, 3.51]), wash hands with soap (aOR = 1.52, 95%CI [1.09, 2.10]) and dry their hands with a dryer (aOR = 1.78, 95%CI [1.16, 2.71]), but they were less likely to always wash their hands after using the toilets (aOR = 0.57, 95%CI [0.32, 1.00]). Among 70 public toilets observed, 9 provided alcohol for toilet seat disinfection, 52 provided soap, 33 provided paper towels, and 41 had working hand dryers.

**Conclusions:**

Despite the overall improvement, residents’ hygiene behaviors in public toilets and the supply of hygiene amenities were still suboptimal during the pandemic. Further hygiene education and an adequate supply of hygiene amenities in public toilets are needed to promote residents’ hygiene behaviors.

## Background

On March 11, 2020, the World Health Organization (WHO) declared the novel coronavirus (COVID-19) outbreak a global pandemic. COVID-19 was first reported in December 2019 in Wuhan, China, and subsequently spread across the country, tragically leading to more than 20 thousand deaths in China by June 2022 [1, 2]. As the virus could be transmitted by infective respiratory droplets and fomites [3], hygiene behavior is undoubtedly one of the essential non-pharmaceutical measures to contain the COVID-19, in addition to an effort to develop an effective vaccine and search for therapeutic agents [4, 5]. During the pandemic, WHO [6] and governments in many countries [7–10] including China published guidance on personal hygiene for the general population to prevent disease transmission.

Public toilets are naturally confined spaces that are ideal breeding grounds of infectious agents [11, 12]. There is a considerable mobile population in close contact in public toilets, and public toilets are risky places of infectious disease transmission [13]. COVID-19 patients and asymptomatic carriers excrete pathogens by coughing, spitting and egestion [3]. The viruses remain on the toilets’ seats after being used by COVID-19 patients and carriers [14]. Studies revealed that massive particles are transported upward when flushing, with substantial quantities of infectious aerosols reaching above the toilet seat to spread the virus [15–18]. In addition, if wiping after toilet use has not been done properly, the pathogens may remain in the hands of the infected person and contaminate all surfaces being touched within the toilet area. These findings mandate the necessity of hygiene behaviors when using toilets (e.g., toilet seat disinfection and cleaning before using, closing the toilet lid when flushing, etc.) during the pandemic.

Moreover, hand hygiene is one of the effective ways to reduce the risks of getting infection during epidemics, including of COVID-19. Washing hands with soap help to remove microbes present on hand, and drying hands prevents transmission of microbes to and from wet hands [19]. In China, handwashing facilities and supplies (e.g., basins and soap) are in public toilets in most public places. Washing hands with soap after using the toilet and drying hands are two key steps recommended by Centers for Disease Control and Prevention (CDC) to prevent transmission [8]. As the infectious virus could be detected on the surface of the toilets after being used by patients with COVID-19 [3], poor hand hygiene behaviors (e.g., not washing hands, not using soap when washing, not drying hands after washing) in public toilets increase the risks of transmission.

To prevent person-to-person transmission, more intensive daily deep cleaning and disinfecting were adopted in public toilets during the pandemic [20]. Nevertheless, public toilet users with undesirable hygiene behaviors are still vulnerable. In late March, 2020, considering the controlled domestic epidemic, the Chinese government started easing the lockdown [21, 22], allowing different sectors to reopen and people accessing public places (i.e., any place to which the public has access, such as shopping malls and parks) [23, 24]. Personal hygiene is especially important to prevent transmission among a larger flow of people in public toilets when resuming normal life activities. We therefore questioned whether hygiene behaviors in public toilets had improved among the residents in Hangzhou in response to the unpredictable domestic outbreaks and the global pandemic.

Raised hygiene knowledge and awareness [25, 26] and higher risk perceptions [27–29] promote hygiene behaviors. During the pandemic, authorities organized pervasive public health education campaigns on personal hygiene to prevent acquisition of the virus [30]. In addition, the pandemic itself increases people’s perceived risks of getting infected [27, 28, 31, 32]. But whether there were improved hygiene awareness and risk perceptions of using public toilets among residents before and during the COVID-19 pandemic remain unknown.

In this study, we compared the data of residents in the same study sites about their toilet hygiene perceptions and practices, collected 1 year before and 1 year after the peak of the COVID-19 epidemic in China (December, 2019 to March, 2020) [33]. This study aimed to establish whether there had been an improvement in residents’ public toilet hygiene awareness and behaviors, and their perceived risks of contracting diseases when using public toilets, with exposure to the COVID-19 pandemic and hygiene-related health education from the government.

## Methods

### Study design and participants

We conducted two rounds of cross-sectional surveys among the residents in Hangzhou, China. Hangzhou is a well-developed city in eastern China, where there is a considerable number of migrant workers and tourists from all parts of the country. According to national data, Hangzhou had more than 6000 public toilets, ranked fourth nationwide in 2018 [34]. The first round of data collection was conducted to evaluate the hygiene perceptions and practices among the Hangzhou residents before the COVID-19 pandemic, between November and December 2018. To evaluate the improvement in the hygiene perceptions and practices during the pandemic, we conducted the second round of data collection between December 2020 and January 2021 when the pandemic was still in place but people’s life almost returned to normal. The target population were individuals over 16 years old residing in Hangzhou. Mandarin is recognized as the official spoken language, while the Hangzhou dialect is a regional spoken language. As our investigators could not speak Hangzhou dialects (i.e., unofficial language), we excluded elderly residents who only understood Hangzhou dialects but not Mandarin. All six major districts in Hangzhou (i.e., Shangcheng District, Xiacheng District, Jianggan District, Gongshu District, Xihu District, Binjiang District), where there are high density of population and public toilets [35], were purposely chosen. We chose two communities in each of six districts from its community lists based on random numbers as our survey sites. We conducted both rounds of data collection at the same survey sites. In each round of data collection, we recruited 20 to 30 residents in each community using a street-intercept method [36] that our research assistants invited passers-by from the study sites to participate in our study. The respondents of two rounds were recruited from the same communities but not the same individuals because we were not able to contact the same participants of the first round of data collection without their contact information. Self-administered paper questionnaires were distributed by our research assistants to the participants. Most participants completed the questionnaires by themselves and handed them back to the research assistants. For some elderly participants who had difficulties reading, our research assistants were on site to support and assist their reading and understanding of the questionnaire.

The questionnaire was developed based on literature review and the qualitative study adapted from our previous studies [37]. In this study, we converted the original questionnaire from the traditional Chinese version (i.e. writing system used in Hong Kong) into the simplified Chinese version (i.e. writing system used in mainland China) and modified several wordings of the questions to fit the cultural context in mainland China. Despite that Hangzhou has mixed settings of public squat and sitting toilets, with preponderance of squat toilets, we decided to keep the questions related to using sitting toilets in the original questionnaires. The questionnaire was pilot-tested by face-to-face interviews with five laymen in Xihu district, Hangzhou, who did not have an education background of medicine, pharmaceutics, biology or public health, and showed good validity. There were 18 different research assistants for each round of data collection. Before data collection, all the research assistants had standard training for 30 minutes concerning how to recruit study participants, how to elaborate the questions when necessary, and how to check the validity of collected questionnaires. In both rounds of data collection, two researchers (JX & XX) were responsible for training and supervision.

We estimated the proportion of having hygiene related behaviors in this study at 70% according to the data from a similar study conducted among the Chinese residents in Hong Kong [37]. Based on the estimate of 70% [37], we set a target effective sample size of 291 for each round of data collection, providing 80% power to detect the ±10% margin of difference, with a two-sided type I error rate of 0.05, according to the sample size formula for comparison of two proportions [38]. The total sample size was adjusted to 364 for each round considering a potential non-response rate of 20% from our prior survey experience. There were 312 and 314 valid questionnaires in the first and second round of data collection, respectively. All participants were informed that their participation was confidential, voluntary, anonymous, and that they could quit at any time. A study compensation worth five RMB (US$ 0.77) was provided as a token of gratitude for their time participating in the study.

We conducted an additional observational study at toilets in different public places located at the study sites (about five public toilets in each community) to record toilets’ provision of hygiene amenities, between February and March 2021, during the second round of data collection. Four trained data collectors (two males and two females) were responsible for observing all of the sampled public toilets. Those who were responsible for managing public toilets might replenish hygiene amenities when they noticed an investigation taking place. Therefore, our data collectors pretended as public toilet users and recorded the data using a cellphone to fill the digital structured checklist immediately upon arrival to avoid the Hawthorne effect [39]. The structured checklist recorded toilets’ provision of hygiene amenities, including paper tissue, alcohol disinfectants, tap water, soap, paper towels, and working hand dryers, as well as types of toilets. The observational study lasted for 2 weeks, as the four data collectors visited five public toilets in one community for 8 h per day on average, from 12 noon to 8 pm. In total, 70 public toilets were observed.

### Measures

During the pandemic, many approaches were adopted to prevent disease transmission among the general population. More intensive cleaning and disinfection were conducted to mitigate the transmission caused by touching contaminated surface in public places [20]. Public places, such as hotels, provide toothpicks for their guests as a tool to press the button on the elevator. Besides, the government provide hygiene amenities and hygiene promotion activities/messages to raise residents’ hygiene awareness and promote hygiene practices during the pandemic. Alcohol disinfectants were distributed to residents, along with advocation of disinfecting their home [40]. Guidance on personal hygiene and health education materials (e.g., pamphlets, posters, booklets) were widely publicized [22, 41].

### Hygiene behaviors in public toilets

Respondents’ hygiene behaviors when using sitting toilets were collected by asking how often they would: (1) clean the toilet seat with alcohol; (2) clean the toilet seat with tissue paper; (3) put tissue paper on the toilet seat before using; (4) flush with the toilet lid closed. Respondents’ hand hygiene behaviors after using the toilet were collected by asking their frequency to (1) wash their hands after using the toilet; (2) wash their hands with soap after using the toilet; (3) dry their hands with paper towels; (4) dry their hands with a dryer. Response choices were “always,” “sometimes,” or “never”. We defined those respondents who chose “sometimes/never” responses as having low compliance with hygiene behaviors, thus they were combined for analysis.

### Hygiene awareness of hygiene behaviors in public toilets

Respondents were asked to choose all of the hygiene behaviors in public toilets that they believed would increase the risk of disease transmission with “Yes” and “No” options among the following choices: (1) touching contaminated toilet facilities; (2) flushing the toilet without the lid closed; (3) not washing one’s hands after using the toilet; (4) not using soap to wash one’s hands after using the toilet; (5) not drying one’s hands after washing them.

### Risk perception of using public toilets

Residents’ perceived risks of getting infected when using public toilets were measured by three items using a 5-point Likert scale ranging from 1 (strongly disagree) to 5 (strongly agree): (1) toilet hygiene has no direct relationship with infectious diseases; (2) worried about getting infected in the public toilet; (3) public toilet is a breeding ground for infectious agents. Residents who chose “4–agree” and “5–strongly agree” were categorized as one group that indicated agreement with the statement; residents who chose “1–strongly disagree,” “2–disagree,” and “3-neutral” were categorized as another group that did not indicate agreement.

### Sociodemographic factors

Sociodemographic factors included gender (male/female), marital status (unmarried/married), age (18–39 years/40–59 years/60 years or older), education level (middle school and under/high school/college and above), and monthly household income (less than ¥5,000/ ¥5,001–¥18,000/ and more than ¥18,000).

### Statistical analysis

Descriptive analysis was conducted to show frequencies and percentages. Chi-squared test was used to evaluate the similarity of sociodemographic characteristics between the respondents in the first and the second rounds. Bivariate analysis was used to compare the differences in hygiene behaviors in public toilets, hygiene awareness, and risk perceptions between the two rounds of data. We used multivariable logistic regressions to evaluate the differences of the residents’ toilet hygiene behaviors/perceptions between the two rounds, with the first-round respondents treated as the reference group. Regression models were adjusted for gender, marital status, age, education level, and household income. All statistical analyses were performed using SPSS 24.0 with the statistical significance set at *p* < 0.05.

## Results

In the first round of data collection, among the 414 residents asked, 316 (76.3%) agreed to participate. Four questionnaires were excluded due to incompleteness or inconsistency in options for two similar questions, resulting in 312 valid questionnaires in total. In the second round of data collection, the response rate was 76.6%, and we got 314 valid questionnaires with 17 discarded. Among the 312 first-round respondents (Table 1), more than half were female (54.5%), married (63.1%), of age group of 18 to 39 years (60.3%), and had a monthly household income between ¥5,001 and ¥18,000 (57.6%); 46.0% had a college or above education level. Similarly, more than half of the 314 second-round respondents were female (51.3%), married (62.7%), aged 18–39 (56.1%), and had a household income between ¥5,001 and ¥18,000 (50.3%); 56.7% had a college or above education level. There were no significant differences in the distributions of demographic characteristics between the two rounds, except education levels (*p* = 0.002).

**Table 1:** Sociodemographic characteristics of the respondents (*n* = 626)

	Round 1	Round 2	χ^**2**^/t test
	**n(%)**	**n(%)**	**p**
**Gender**			0.421
Male	142(45.5)	153(48.7)	
Female	170(54.5)	161(51.3)	
**Marital status**			
Unmarried	115(36.9)	117(37.3)	0.917
Married	197(63.1)	197(62.7)	
**Age**			0.243
18–39	187(60.3)	176(56.1)	
40–59	91(29.4)	92(29.3)	
≥60	32(10.3)	46(14.6)	
**Education level**			0.002
Middle school and under	76(24.4)	80(25.5)	
High school	92(29.6)	56(17.8)	
College and above	143(46.0)	178(56.7)	
**Monthly household income**			0.119
< ¥5,000 ($725)	78(25.1)	101(32.2)	
¥5,001–¥18,000 ($725–$2610)	179(57.6)	158(50.3)	
> ¥18,000 ($2610)	54(17.4)	55(17.5)	

Table 2 presents differences of hygiene behaviors in public toilets between the two rounds of respondents. Compared with those of the first-round, the second-round respondents reported a higher proportion of always cleaning toilet seat with alcohol (5.5% vs. 10.2%, *p* = 0.031); though there were no differences in proportions of cleaning the toilet seat with paper (45.9% vs. 40.4%, *p* = 0.168), putting paper on the toilet seat before using (33.6% vs. 29.6%, *p* = 0.292), or flushing with the toilet lid closed (36.7% vs. 31.6%, *p* = 0.182). Respondents from both rounds of data collection reported high proportions of always washing their hands after using the toilet, with the preponderance of those from the first round (92.9% vs. 87.6%, *p* = 0.026). Respondents from the second round of data collection reported a higher proportion of always washing their hands with soap after using the toilet (43.0% vs. 51.9%, *p* = 0.026) and drying their hands with a dryer (14.4% vs. 22.9%, *p* = 0.006) than those from the first round, with no differences in proportions of reporting always drying hands with paper towels (54.4% vs. 54.1%, *p* = 0.949). Multivariate logistic regressions suggest stable differences between the two rounds, that the second-round respondents were more likely to always clean their toilet seat with alcohol (aOR = 1.88, 95%CI [1.01, 3.51]), wash their hands with soap after using the toilet (aOR = 1.52, 95%CI [1.09, 2.10]), and dry their hands with a dryer (aOR = 1.78, 95%CI [1.16, 2.71]), while they were less likely to always wash their hands after using the toilet (aOR = 0.57, 95%CI [0.32, 1.00]).

**Table 2:** Changes in public toilet using behaviors among the respondents (*n* = 626)

	Round 1	Round 2	χ^**2**^ test	Multivariable logistic regression	
	Always n(%)	Always n(%)	p	Adjusted odds ratio (95% CI)^**a**^	p
Clean the toilet seat with alcohol	17(5.5)	32(10.2)	0.031	1.88(1.01,3.51)	0.047
Clean the toilet seat with tissue paper	141(45.9)	127(40.4)	0.168	0.83(0.60,1.15)	0.263
Put tissue paper on the toilet seat before using	103(33.6)	93(29.6)	0.292	0.87(0.62,1.24)	0.447
Flush with the toilet lid closed	112(36.7)	99(31.6)	0.182	0.80(0.57,1.12)	0.199
Wash your hands after using the toilet	287(92.9)	275(87.6)	0.026	0.57(0.32,1.00)	0.048
Wash your hands with soap after using the toilet	132(43.0)	163(51.9)	0.026	1.52(1.09,2.10)	0.013
Dry your hands with paper towels	167(54.4)	170(54.1)	0.949	1.03(0.75,1.43)	0.845
Dry your hands with a hand dryer	44(14.4)	72(22.9)	0.006	1.78(1.16,2.71)	0.008

^a^Reference group: Round 1; adjusted odds ratios adjusted for gender, marital status, age, education levels, and monthly household income levels

During the pandemic, respondents reported poor hand hygiene awareness: less than half of respondents reported hygiene awareness of risks of flushing the toilet without the lid closed (29.9%), not using soap to wash one’s hands (43.6%), and not drying one’s hands (21.3%). A higher proportion of the second-round respondents than the first-round respondents believed that touching contaminated toilet facilities (70.2% vs. 77.4%, *p* = 0.041) (Table 3), not using soap to wash one’s hands after using the toilet (29.8% vs. 43.6%, *p* < 0.001), and not drying one’s hands after washing (15.1% vs. 21.3%, *p* = 0.042) could increase the risks of getting infected in a public toilet. There were no differences in hygiene awareness of flushing with lid closed (26.9% vs. 29.9%, *p* = 0.403) and hand washing (77.6% vs. 75.2%, *p* = 0.479) between the two rounds. The differences of hygiene awareness between two rounds remained significant in the multivariate regression models, that the second-round respondents had higher odds of believing that touching contaminated toilet facilities (aOR = 1.72, 95%CI [1.17, 2.54]) and not using soap to wash one’s hands after using the toilet (aOR = 1.93, 95%CI [1.38, 2.72]) could increase the risks of getting infected in a public toilet.

**Table 3:** Changes in hygiene awareness of public toilet using behaviors among the respondents (*n* = 626)

	Round 1	Round 2	χ^**2**^ test	Multivariable logistic regression	
Following behaviors will increase the risk of disease transmission:	Agree n(%)	Agree n(%)	p	Adjusted odds ratio (95 CI%)^**a**^	p
Touching contaminated toilet facilities	219(70.2)	243(77.4)	0.041	1.72(1.17,2.54)	0.006
Flushing the toilet without the lid closed	84(26.9)	94(29.9)	0.403	1.17(0.82,1.67)	0.386
Not washing one’s hands after using the toilet	242(77.6)	236(75.2)	0.479	0.89(0.61,1.30)	0.541
Not using soap to wash one’s hands after using the toilet	93(29.8)	137(43.6)	< 0.001	1.93(1.38,2.72)	< 0.001
Not drying one’s hands after washing them	47(15.1)	67(21.3)	0.042	1.40(0.92,2.13)	0.120

^a^Reference group: Round 1; adjusted odds ratios adjusted for gender, marital status, age, education levels, and monthly household income levels

Bivariate analysis showed that a smaller proportion of the second-round respondents thought that toilet hygiene has no direct relationship with infectious diseases (55.0% vs. 46.2%, *p* = 0.027), and that a higher proportion of them was worried about getting infected in the public toilet (69.8% vs. 79.9%, *p* = 0.003) than those from the first round. However, there were no differences in the proportions of believing public toilet was a breeding ground for infectious agents between the two rounds (60.8% vs. 67.8%, *p* = 0.068) (Table 4). Further regression models indicate that, compared with the first-round respondents, the second-round respondents were less likely to believe there was no direct relationship between toilet hygiene and infectious diseases (aOR = 0.67, 95%CI [0.49, 0.93]) and were more likely to be worried about getting infected when using public toilets (aOR = 1.77, 95%CI [1.20, 2.60]).

**Table 4:** Changes in risk perceptions of using public toilets among the respondents (*n* = 626)

	Round 1	Round 2	χ^**2**^ test	Multivariable logistic regression	
	Agree n(%)	Agree n(%)	p	Adjusted odds ratio (95 CI%)^**a**^	p
Toilet hygiene has no direct relationship with infectious diseases	170(55.0)	145(46.2)	0.027	0.67(0.49,0.93)	0.016
Worried about getting infected in the public toilet	217(69.8)	251(79.9)	0.003	1.77(1.20,2.60)	0.004
Public toilet is a breeding ground for infectious agents	188(60.8)	213(67.8)	0.068	1.33(0.95,1.87)	0.096

^a^Reference group: Round 1; adjusted odds ratios adjusted for gender, marital status, age, education levels, and monthly household income levels

We investigated a total of 70 public toilets, which cover toilets in different public places (malls, streets, restaurants, etc.), with 38 being ladies’ toilets (Table 5). Among all the public toilets, 33 of them (47.1%) had sitting toilets; 35 (50.0%) provided paper tissue and only 9 (12.9%) provided alcohol for toilet seat cleaning and disinfection. Considering handwashing amenities, 69 of them (98.5%) had tap water supply, 52 (74.3%) had soap supply, 33 (47.1%) provided paper towels and 41 (58.6%) had working hand dryers.

**Table 5:** Provision of hygiene amenities in public toilets at the study sites (*n* = 70)

	Shangcheng ***n*** = 8	Xiacheng ***n*** = 15	Jianggan ***n*** = 11	Gongshu ***n*** = 9	Xihu ***n*** = 13	Binjiang ***n*** = 14	Total n(%)
**Public places**							
Shopping malls, supermarkets	4	0	2	3	5	4	18(25.7)
Parks, courts	0	4	0	2	2	0	8(11.4)
Wet markets, grocery markets	0	0	0	0	0	2	2(2.9)
Stadiums	0	1	0	0	0	0	1(1.4)
On the street	2	2	4	4	3	0	15(21.4)
Hotels	0	0	2	0	0	2	4(5.7)
Restaurants	2	2	2	0	2	2	10(14.3)
Cinema, museums, exhibition halls	0	5	0	0	0	2	7(10.0)
Subway, railway stations, bus stations	0	1	1	0	1	2	5(7.1)
**Gender**							
Gents	4	7	5	4	4	7	31(44.3)
Ladies	4	8	6	5	8	7	38(54.3)
Gender neutral	0	0	0	0	1	0	1(1.4)
**Toilet type**							
Both	1	7	4	8	4	3	27(38.5)
Only sitting toilets	1	0	2	0	2	1	6(8.6)
Only squat toilets	6	8	5	1	7	10	37(52.9)
**Supply of hygiene amenities**							
Paper tissue	6	3	8	3	9	6	35(50.0)
Alcohol disinfectant	2	1	1	0	2	3	9(12.9)
Tap water	8	15	10	9	13	14	69(98.5)
Soap	6	9	11	7	10	9	52(74.3)
Paper towels	5	4	7	1	9	7	33(47.1)
Working hand dryers	4	7	7	7	7	6	41(58.6)

## Discussion

This study is the first to compare residents’ perceptions and practices of public toilet using in China before and during the COVID-19 pandemic. Compared with residents before the pandemic, residents during the pandemic were more likely to disinfect the toilet seat, wash hands with soap after using the toilet and dry their hands with a dryer. Residents reported higher risk perceptions of using public toilets and elevated risk awareness of certain unhygienic behaviors (i.e., touching contaminated toilet facilities, not using soap to wash one’s hands after using the toilet, and not drying one’s hand after washing). Even during the pandemic, residents’ hygiene behaviors when using public toilets were still suboptimal, such as toilet seat cleaning and disinfection, flushing with the toilet lid closed, washing hands with soap, and hand drying.

Based on our study, residents showed improved hygiene behaviors related to disinfection (e.g., cleaning the toilet seat with alcohol, washing hands with soap) during the pandemic. There was more intensive daily disinfection in public toilets during the pandemic [42]. The doubled rate of seat disinfection when using public toilets may be due to alcohol disinfectant distribution for residents by many local governments and advocation of alcohol disinfectant use at home during the pandemic [40]. However, the proportions of toilet seat disinfection were extremely low in both rounds. An inadequate supply of alcohol disinfectants in public toilets may partly lead to the low proportion of toilet seat disinfection.

A higher proportion of residents reported washing hands with soap, as well as drying their hands with a hand dryer. During the pandemic, the Chinese government developed health education materials to emphasize the significance of hand hygiene for preventing transmission of COVID-19, and recommend steps to wash hands appropriately [22, 41]. According to our results, residents reported better hygiene awareness of washing hands with soap during the pandemic. Besides elevated awareness, increased risk perception during the pandemic is another facilitator of hand hygiene behaviors [28]. However, the proportions of washing hands with soap (51.9%) and drying hands after washing hands (with paper towels or a dryer, 56.4%) only reached about half in the second round, and merely 39.5% of the residents both washed hands with soap and dried their hands. The suboptimal hand hygiene behaviors may be partly explained by raised but still poor hand hygiene awareness (less than 50%) during the pandemic found in our study. In addition, an inadequate supply of handwashing amenities is another barrier to hand hygiene, no matter whether they are aware of the necessity of these hygiene behaviors or not. Thus, more intensive health education on hand hygiene and adequate handwashing amenities (e.g., soap, paper towels, hand dryers, and even sanitizers) should be provided in public toilets to encourage better toilet hygiene.

Residents reported increased risk perceptions of using public toilets during the pandemic, which partly contributed to residents’ improved hygiene behaviors mentioned above. Increased risk perceptions of infectious diseases were associated with better adoption of preventive behaviors both in daily life [43, 44] and during epidemics [45–47]. Health education that emphasizes the effectiveness of maintaining hygiene behaviors to prevent infection, such as related slogan posted in public toilets, distributed health education materials, and media publicity [22, 41], may motivate and shape residents’ hygiene behaviors during pandemic periods.

To mitigate the transmission caused by touching contaminated surface in public places, many approaches were adopted, such as more intensive cleaning and disinfection [42]. During the pandemic, residents reported increased awareness of the risks of touching contaminated toilet facilities. The concern of touching contaminated surfaces when using manual faucets may be a barrier against hand washing during the pandemic. As automatic faucets enable users to wash hand without touching contaminated water tap, it is recommended that public toilets be equipped with more automatic faucets to promote handwashing behavior, especially during the pandemic. In addition to handwashing behavior, residents’ behavior of flushing the toilets with the lid closed decreased slightly, which might also be explained by their unwillingness to touch the contaminated surfaces (i.e., toilet lids). Thus, more pervasive health education on adherence to hygiene at the cost of touching contaminated surfaces (i.e., closing the toilet lid when flushing and hand washing after using the toilet) is needed.

### Limitations

Our study has several limitations. Firstly, residents’ hygiene behaviors when using public squat toilets were not included in this study, and it deserves future investigations considering the prevalence of public squat toilets in China. Nevertheless, behaviors included in this study are applicable to reflect changes in residents’ hygiene behaviors when using public toilets during the pandemic. Secondly, convenience sampling methods limited the representativeness. To improve the representativeness, we maintained balanced sociodemographic distributions and used districts as stratifiers. We recruited different study participants during the two rounds of data collection. However, we recruited them at the same study sites. We trained all research assistants before the data collection with limited time. Besides, our study was conducted in a limited geographical area, and it is uncertain whether our findings can be generalized to a broader population. As residents all over China were profoundly impacted by the COVID-19 pandemic and were all exposed to hygiene-related health education from the government, the changes found among the residents in Hangzhou may reflect the situation of the general population in China to some extent. Moreover, self-reported questionnaires could induce social desirability. Nevertheless, considering both samples were prone to overestimate their hygiene behaviors, comparisons between the two samples may somehow counteract this bias. The study did not include observation of reported behaviors to confirm what was reported, which is considered a gold standard to measure hygiene behaviors. Finally, this study did not present data on reasons why participants did not perform certain hygiene behaviors (e.g., closing the toilet seat when flushing). Further qualitative studies were required for explanations.

## Conclusions

During the COVID-19 pandemic, residents in Hangzhou showed better hygiene behaviors, such as cleaning the toilet seat with alcohol, washing hands with soap, and drying hands with a dryer, though a slightly lower proportion of them washed hands after using the toilet (probably due to not wanting to touch the water tap). Residents’ raised awareness of hygiene behaviors and increased risk perception of disease transmission when using public toilets contribute to improved hygiene behaviors during the pandemic. Despite the overall improvement, residents’ certain hygiene behaviors when using toilets (toilet seat disinfection and cleaning before using and flushing with the toilet lid closed) and hand hygiene behaviors (washing with soap and hand drying) were still unsatisfactory. Good hygiene practices in public toilets are not only efficient to contain the transmission of new infectious diseases, but also helpful to prevent outbreaks of common infectious diseases, such as influenza epidemics, outbreaks of norovirus. More efforts are needed from the government to improve suboptimal behaviors including but not limited to those found in this study. Further hygiene education and an adequate supply of hygiene amenities in public toilets are needed to promote residents’ hygiene behaviors.

## Data Availability

The datasets generated and/or analysed during the current study are not publicly available due to the fact that our colleagues are now analyzing this data for other papers and it is not appropriate to make the raw data publicly available but are available from the corresponding author on reasonable request.
